# Editorial: Therapeutic Targeting of Cancer Stem-Like Cells (CSC) – The Current State of the Art

**DOI:** 10.3389/fonc.2020.00243

**Published:** 2020-02-28

**Authors:** Cyril Corbet, Alexandre Prieur

**Affiliations:** ^1^Pole of Pharmacology and Therapeutics (FATH), Institut de Recherche Expérimentale et Clinique, UCLouvain, Brussels, Belgium; ^2^ECS-Progastrin Chemin de la Meuniere 12, Prilly, Switzerland

**Keywords:** cancer stem cells (CSC), therapeutic targeting, antibodies, transcription factors, small molecules

Cancer stem-like cells (CSC) represent a small population of tumor cells that are thought to exhibit a tumor-initiating potential, as well as enhanced therapy-resistant and metastasis-forming capacities, thereby actively contributing to clinical relapse and poor prognosis in cancer patients. Although all these phenotypic properties make CSC targets of great interest in drug discovery, there are currently only few therapeutic approaches that have reached late stages of clinical development in oncology. This Special Topic of Frontiers in Oncology attempts to address some major concerns related to the therapeutic targeting of CSC: what are these cells, how may the specific permissive microenvironment (the so-called stem cell niche) be therapeutically exploited, and what are the emerging therapeutic avenues aiming to eradicate this specific tumor cell subpopulation.

The papers in this Special Topic can be categorized into three main parts. The first part highlights the influence of tumor microenvironment (TME) peculiarities on the emergence and/or maintenance of stem-like phenotypes and how this can be therapeutically integrated and exploited. This subject is broadly reviewed by Sun et al. and then also addressed by De Angelis et al. in the specific contexts of cell dormancy and therapy resistance. Chan et al. discuss the interplay between CSC and stromal cells, including cancer-associated fibroblasts and tumor-infiltrating mesenchymal stem cells (MSC). Avnet et al. also summarize the currently available preclinical models that help evaluate the functional interaction between MSC and cancer cells. Finally, Vander Linden and Corbet describe how tumor acidosis, a common hallmark of TME in solid tumors, may provide a permissive niche to shape more aggressive stem-like cancer cell phenotypes. These different articles also review new therapeutic options aiming to eradicate CSC by integrating and/or exploiting the TME niches in order to overcome therapy resistance and metastatic dissemination.

The second set of papers relates to the metabolic preferences of CSC and emerging metabolism-based therapeutic strategies that are currently in (pre)clinical testing for cancer treatment. Jagust et al. make an overview of metabolic pathways that support CSC phenotypes in different cancer types, while Garnier et al. discuss specifically about the metabolism of glioblastoma stem-like cells and its role in tumor progression and clinical relapse. Recalcati et al. report an increasing evidence for dysregulated iron homeostasis in cancer cells, with a special focus on liver CSC. These authors also discuss new therapeutic options aiming to manipulate iron metabolism for anti-tumor therapy. Another review article from Lucena-Cacace et al. reports the important role for nicotinamide phosphoribosyltransferase (NAMPT), the rate-limiting enzyme in the NAD^+^ salvage pathway, in the maintenance of a glioma cancer stem-like cell (GSC) population. They discuss how NAD^+^ homeostasis supports metabolic and non-metabolic processes that contribute to a GSC phenotype. Finally, Han et al. report, in an research article, that radioresistant breast cancer (stem) cells rely on fatty acid metabolism to survive and grow, with carnitine palmitoyl transferases 1A and 2 as main actors and potential therapeutic targets.

The third and final part of this special issue is focused on new therapeutic avenues to target CSC populations. Marcucci et al. discuss the potential application of antibody-drug conjugates as tools for a selective eradication of CSC and how some limitations related to their use may be addressed. Roth et al. describe the roles of the renin-angiotensin system (RAS) in CSC biology and how RAS modulators may offer new therapeutic approaches to target CSC and reduce tumor growth. Finally, Civenni et al. review the influence of transcriptional regulators in the emergence and/or maintenance of a stem-like cell population in the context of prostate cancers, and describe current therapeutic strategies aiming to interfere with specific transcriptional programs and associated stem-like phenotypic changes.

## Author Contributions

CC has written the editorial. CC and AP have collected and edited the articles of the Research Topic.

### Conflict of Interest

The authors declare that the research was conducted in the absence of any commercial or financial relationships that could be construed as a potential conflict of interest.

